# Unveiling the intricacies of gene delivery: Caveolae-mediated endocytosis induces efficient mRNA delivery in slow-dividing cells

**DOI:** 10.1016/j.omtn.2023.07.024

**Published:** 2023-08-10

**Authors:** Sabine den Roover, Joeri L. Aerts

**Affiliations:** 1 Neuro-Aging & Viro-Immunotherapy (NAVI) Research Group, Vrije Universiteit Brussel, Brussels, Belgium

**Figure 1:**
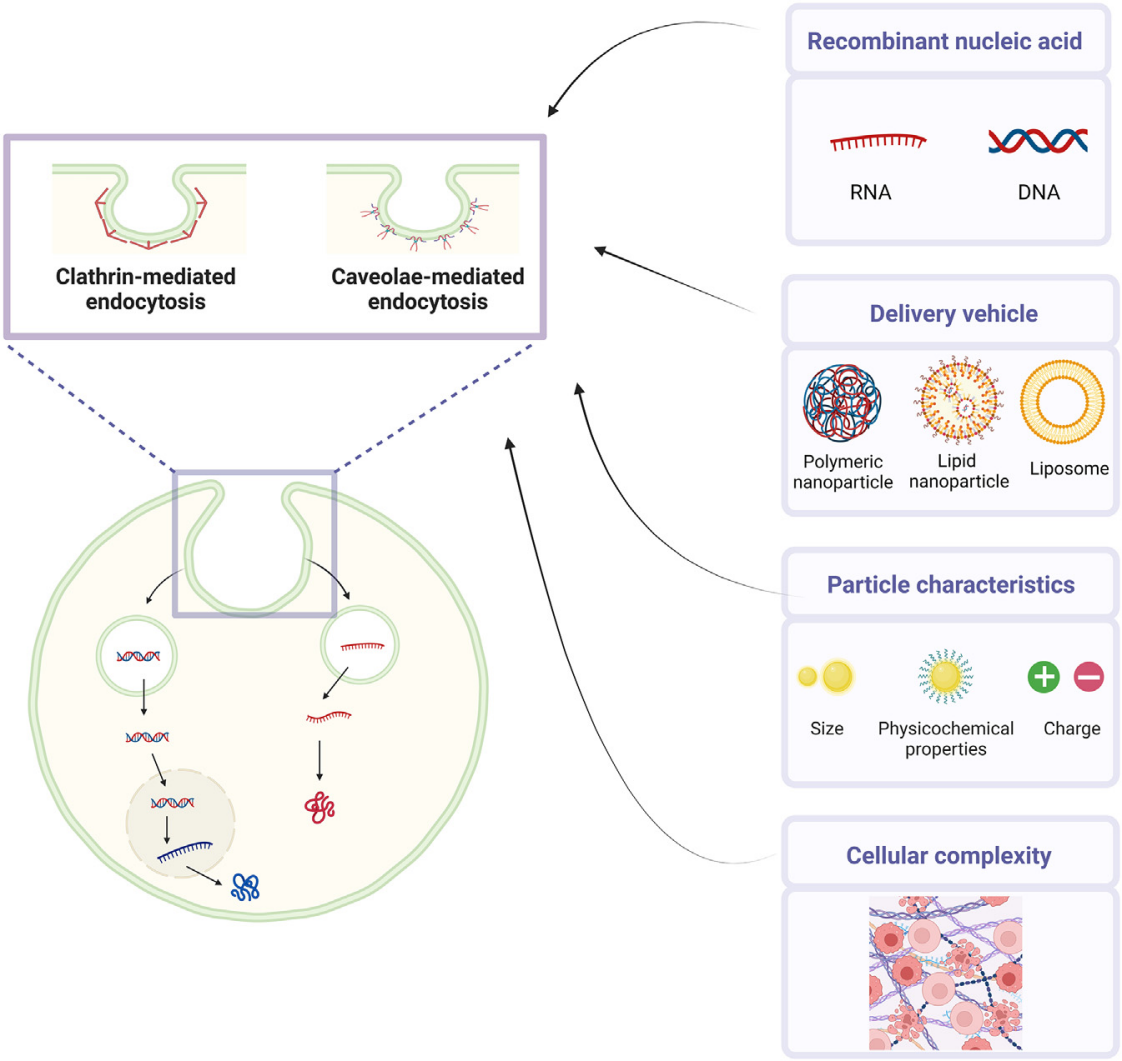
Factors influencing the delivery of nucleic acids and the expression of the transgene in target cells.

In order to unlock the full potential of gene therapy, understanding the cellular uptake mechanisms and choosing the most suitable delivery vehicle are crucially important. In the paper by Del Toro Runzer and colleagues recently published in *Molecular Therapy – Nucleic Acids*, the roles of the cellular uptake mechanism, microenvironment, and cell type on the delivery of genetic materials were extensively investigated (Figure 1).^1^ These insights could not only lead to improved delivery but also to reduced cytotoxicity and beneficial therapeutic outcomes.

Over 5 decades ago, Friedmann and Roblin proposed that inherited genetic disorders can be treated by the introduction of a functional gene copy.^2^ The most widely used approaches for gene therapy are viral vectors and recombinant nucleic acids. Plasmid DNA (pDNA) was the first nucleic acid to be used as a therapeutic molecule because of the natural capacity of bacteria to amplify plasmid vectors to large copy numbers, allowing easy upscaling and, because of the high intrinsic stability of pDNA, affording long-term storage without an extensive cold chain. Even though the use of pDNA vector in small animals was quite promising, results in large animals and humans turned out to be rather disappointing as important limitations were observed, including poor intracellular bioavailability and recombination with host genomic DNA, giving rise to ethical considerations and hindering their clinical application.^3^ More recently, owing to the success of COVID-19 vaccines, messenger RNA (mRNA) has gained a lot of interest as an alternative gene delivery method. Unlike pDNA, mRNA does not represent a risk for off-target effects, is only transiently expressed, and does not require transport across the nucleus. Nonetheless, important drawbacks for mRNA also need to be considered. Thus, it is extremely sensitive to ambient RNases and therefore requires a suitable formulation platform. Moreover, unmodified mRNA is easily recognized by various innate immune sensors including endosomal Toll-like receptors and cytoplasmic sensors such as RIG-I and MDA-5. This initiates strong immune responses, including the production of inflammatory cytokines (IL-6, TNF) and type I interferon, which hinder successful gene therapy. To address these challenges, researchers have focused on designing novel delivery vehicles, resulting in efficient gene delivery without excessive immune activation.

Throughout the years, it has been clear that gene therapy has a broad application potential across various fields of medicine such as regenerative medicine and the treatment of genetic diseases and cancer. For example, therapeutics for bone tissue regeneration require constant advancement to find efficient and cost-effective treatment modalities. Medtronic’s Infuse is one of the first recombinant human bone morphogenetic proteins (using rhBMP-2) used in regenerative medicines. Despite being FDA approved, the clinical efficacy of this therapy remains limited, and its use is associated with important adverse effects.^4^ In 2022, Balmayor et al. evaluated the ability of chemically modified mRNA (cmRNA) encoding rhBMP-2 to heal a segmental defect in the rat femur. Various approaches were hereby considered, including the design of a suitable delivery vehicle (lipoplex based), stable nucleotide-modified mRNA (cmRNA) encoding BMP-2, and a suitable *in vitro* model.^5^^,^^6^^,^^7^ While the proposed applications were promising, several challenges such as the delivery route, cellular environment complexity, and selection of an appropriate delivery vehicle need to be overcome. To ensure efficient gene delivery, it is important to understand the cellular uptake mechanism and efficiencies of these genetic materials as these components are likely to influence the intracellular fate and biological response of the therapeutic molecule.

Del Toro Runzer et al. compared the cellular uptake and transfection efficiency between pDNA and mRNA complexes. For this purpose, several human primary cell types relevant to the field of tissue engineering and regenerative medicine were used as *in vitro* models.^1^

Three different commercially available transfection agents, Lipofectamine 3000, 3D Fect, and Trans-IT-X2 were compared. In addition to their compatibility with both pDNA and cmRNA, these agents differ from each other in size and chemical composition. Thus, both Lipfectamine 3000 and 3D Fect are lipid-based, whereas Trans-IT X2 is a polymer-based transfection reagent, consisting of cationic polymers. Initially, three different pharmacological inhibitors, chlorpromazine, wortmannin, and genistein, were applied to determine the uptake mechanism of each of these transfection agents. Generally, two main entry mechanisms, can be considered: endocytosis and non-endocytic mechanisms. Endocytosis-dependent entry mechanisms include phagocytosis, clathrin-mediated endocytosis, caveolin-mediated endocytosis, clathrin/caveolae-independent endocytosis, and micropinocytosis. Interestingly, the cellular uptake results were in line with the endocytic pathways that could be predicted by the size of the particles. Small particles (±200 nm) are typically taken up via clathrin-mediated endocytosis, while larger particles (±500 nm) are taken up via caveolae-mediated endocytosis. Indeed, both clathrin- and caveolin-mediated endocytosis were equally involved in the uptake of the lipoplexes, while polymer complexes rely on caveolae-mediated endocytosis. Even though the selected inhibitors largely cover the different endocytosis-dependent pathways, other targeting molecules such as pimozide (phagocytosis) could be explored.^8^ Choosing a suitable delivery system greatly affects the transfection efficiency. In addition to these commercially available agents, several novel nanoformulations have been developed including lipid nanoparticles, used extensively in the mRNA-based COVID-19 vaccines, and layer-by-layer polyelectrolyte particles. These should also be considered and investigated in the future.^9^^,^^10^

Developing an effective therapy for tissue engineering applications requires knowledge of the interaction of the nanoformulation with specific target cells as well as with the cellular microenvironment. Nucleic acid delivery in three relevant human *in vitro* models, mesenchymal stromal cells, fibroblasts, and osteoblasts, was compared by Del Toro Runzer et al. Interestingly, cmRNA is readily taken up and translated in slow to non-dividing cells such as osteoblasts as opposed to pDNA, which was more efficient in fast-dividing fibroblasts. Furthermore, a unique 3D nanofibrous scaffold was used to imitate the structure of the extracellular matrix. Compared to the 2D model, both pDNA and mRNA showed an increased protein translation in the 3D scaffold. These findings highlight the importance of using a physiological resemblance model. Although the authors aim to apply this system to tissue engineering and bone reconstitution therapy, a broader application potential could be envisioned. One might thus wonder whether similar outcomes will be obtained in other target cells such as dendritic cells and human primary T cells. For instance, it has been shown that the complexity of the tumor microenvironment hinders the delivery of the mRNA-based vaccines into target cells. Additional studies on developing the most suitable delivery system to circumvent this and other hurdles should be considered.

In conclusion, Del Toro Runzer et al. nicely demonstrate that the choice of nucleic acids along with their delivery system and the cellular complexity significantly impact the effectiveness of gene delivery. Future investigations exploring the use of alternative nanoformulations, such as lipid nanoparticles, as well as relevant cellular models should, however, be conducted. This knowledge is not only of great importance in the field of gene therapy, but can also be expanded to other research fields such as mRNA-based vaccines for cancer and infectious diseases.
